# Mixed High-Risk Endometrial Carcinoma Initially Presented With Atypical Squamous Cells of Undetermined Significance (ASC-US) Cytology: Lessons to Be Learned

**DOI:** 10.7759/cureus.49457

**Published:** 2023-11-26

**Authors:** Chrysoula Margioula-Siarkou, Emmanouela-Aliki A Almperi, Aristarchos Almperis, Konstantinos Dinas, Stamatios Petousis

**Affiliations:** 1 2nd Department of Obstetrics and Gynaecology, Gynaecologic Oncology Unit, Ippokrateio General Hospital of Thessaloniki, Aristotle University of Thessaloniki, Thessaloniki, GRC; 2 2nd Department of Obstetrics and Gynaecology, Gynaecologic Oncology Unit,, Ippokrateio General Hospital of Thessaloniki, Aristotle University of Thessaloniki, Thessaloniki, GRC

**Keywords:** lymphadenectomy, cervical cancer, immunohistochemistry, cytology, endometrial cancer

## Abstract

Endometrial carcinoma represents the most common gynecologic malignancy, affecting mainly postmenopausal women. Early detection and proper management may allow not only a successful treatment but also an acceptable quality of life. Although its usual clinical manifestations, such as vaginal bleeding or a mass stuck out of the cervix, often arouse medical concern, in some cases, there is a dilemma regarding the differential diagnosis of endocervical cancer. Additionally, tumors arising from and confined to the uterine isthmus may often pose a greater diagnostic challenge. Defining the primary origin of the carcinoma is of paramount importance as the treatment plan may be widely different in these two scenarios. Magnetic Resonance Imaging (MRI), markers, and biopsy with immunohistochemistry could facilitate the diagnostic process, but the final diagnosis may even be made from the final surgical specimen in such demanding cases. We present the case of a mixed-type, stage IIIC2, endometrial carcinoma arising from the lower uterine segment initially suspected from atypical squamous cells of undetermined significance (ASC-US) cytology.

## Introduction

Endometrial cancer is the most commonly diagnosed malignancy of the female genital tract. It may originate from the endometrium of the uterine corpus or fundus and the lower uterine segment (LUS), which represents a clinical entity with specific particularities. A LUS carcinoma is a rare tumor that accounts for 3-6% of all cases of endometrial cancer. It expands macroscopically from the isthmus of the uterus through the upper cervix. Due to the location of this carcinoma, it displays histological characteristics of both endometrium and uterine cervix in the glandular epithelium and interstitium. However, the LUS tends to respond poorly to hormone stimulation due to the thin mucosal layer of the endometrium compared to that of the main uterine body. Furthermore, both endocervical and endometrial carcinoma can be manifested clinically with similar symptoms, such as an enlarged cervix, a mass bulging through the cervix, or vaginal bleeding. As a consequence, in cases where both the LUS and the cervix are involved in adenocarcinoma, the condition poses a great challenge to determine the primary origin, even after pathological evaluation of a biopsied specimen [1].

Even though surgery is involved in the treatment regimen of all stages of endometrial cancer, it is indicated only in early-stage cervical cancer, while chemoradiation may be the treatment of choice in advanced-stage cervical cancer. In addition, patients with endometrial cancer with cervical involvement may benefit from postoperative radiation to decrease the risk of local recurrence and improve overall survival [2]. Although diagnostic tools such as Magnetic Resonance Imaging (MRI), hormone receptor immunohistochemistry, and human papillomavirus (HPV) in situ hybridization are useful for distinguishing endocervical and endometrial adenocarcinomas, in most cases described by the literature, the final histological diagnosis may be made even in the final histopathological specimen of hysterectomy. An abnormal result in a cytology test leading to a diagnosis of endometrial cancer in the uterine isthmus is a relatively rare clinical scenario of significant clinical interest.

We present the case of a 53-year-old woman finally diagnosed with stage IIIC2 serous-mixed endometrial carcinoma arising from uterine isthmus that was admitted initially only with atypical squamous cells of undetermined significance (ASC-US) result in cytology test.

## Case presentation

A 53-year-old woman was presented to our department with a histopathological diagnosis of “high-grade invasive carcinoma of unknown primary origin which needs an immunohistochemistry process” asking for further consultation and management. The obstetrical history of the patient included two uncomplicated pregnancies delivered by a caesarian section. Regarding gynecological history and specifically risk factors for HPV infection and cervical disease, the patient reported starting sexual contact at 20 years, overall two previous sexual partners, no smoking habit, not regular condom usage, and no vaccinations against HPV.

The woman had been diagnosed with ASC-US cytology in November 2020, after performing a regular Papanicolaou (Pap) smear examination, without reporting specific symptoms such as vaginal bleeding or anything relevant. No HPV testing had been performed. She was initially advised by the private physician to take vitamins and repeat the cytology test in three months. The patient followed the medical advice, cytology was repeated in February 2021 and a diagnosis of atypical squamous cells (ASC-H) was made. The patient underwent colposcopy with cervical biopsy, which indicated the aforementioned diagnosis of "high-grade invasive carcinoma of unknown origin" for which she was presented in our department.

Clinical findings and diagnostic assessment

At the Gynecologic Oncology Unit of our Department, all cases treated are discussed in our Multidisciplinary Tumour Board (MTB) as per protocol. Since the origin of the tumor was not known and a review of the histopathologic specimen was not possible because of the patient’s disagreement to perform any contact with the previous laboratory and doctor, the decision to perform a pelvic MRI, CT scan of the thorax, upper and lower abdomen and thereafter decide on the therapeutic plan was made. The MRI described a tumor of 35 mm maximal diameter located in the limit between the uterine isthmus and cervix. CT scan indicated no evidence of distant metastatic disease, apart from the pelvic tumor described in the pelvic MRI. Regarding the potential of pelvic node invasions, MRI and CT scans indicated the presence of pelvic nodes of a maximal diameter of 11 mm without being definitive for macrometastatic invasion. The decision of cervical conization and endometrial curettage was made to achieve an accurate diagnosis of tumor origin, while a concomitant laparoscopic pelvic lymph node dissection (PLND) was decided as an initial staging step in case of cervical cancer diagnosis.

Therapeutic intervention

**Figure 1:**
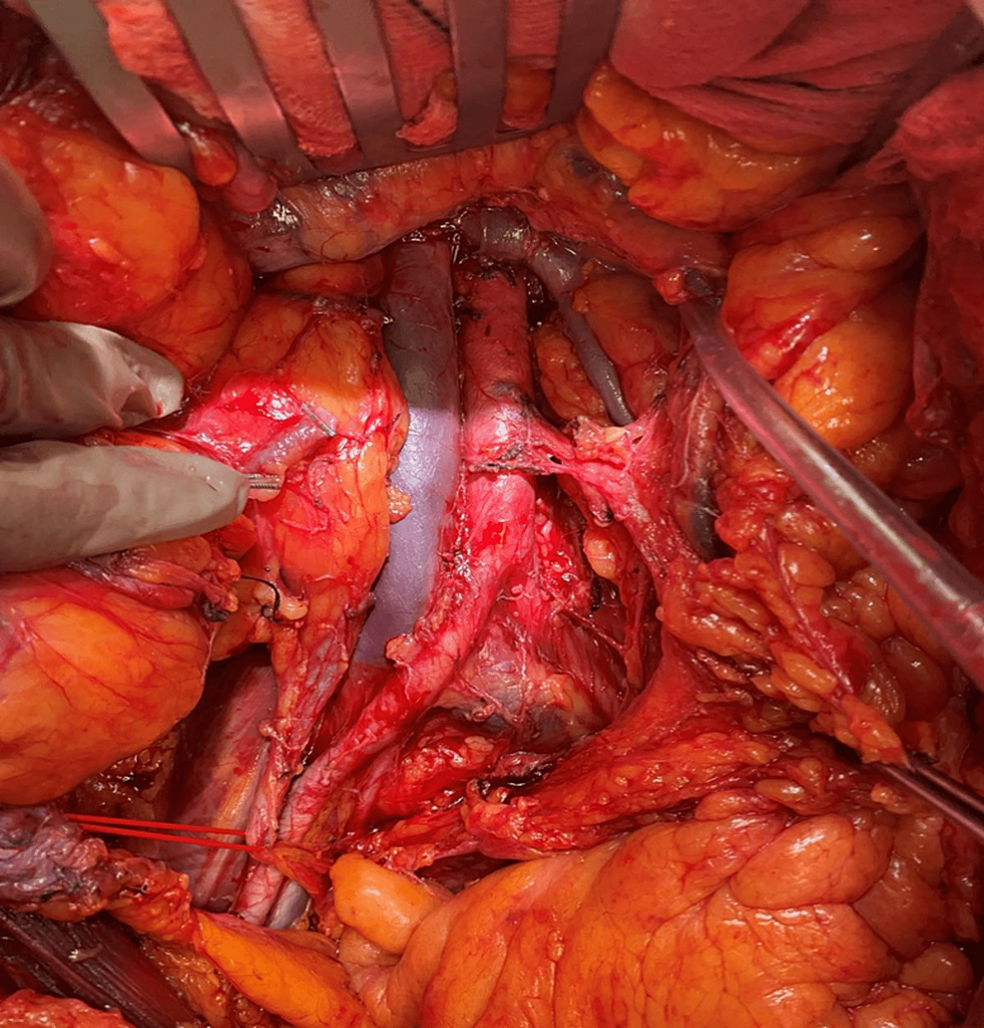
Final surgical anatomy after paraaortic lymphadenectomy The aorta, vena cava, lower mesenteric artery, and left renal vein are being exposed as part of the final surgical anatomy.

The conization resulted in delivering an intact specimen measuring 2 x 1.8 x 0.9 cm with a maximum depth of invasion of 4 mm. The neoplastic cells were p16, p53, CD10, vimentin positive, and p40 negative. No endometrial cancer was impressively detected from the endometrial curettage. But, there were seven of 30 pelvic lymph nodes invaded. According to histopathology and immunohistochemistry, the diagnosis of a uterine carcinosarcoma derived from uterus isthmus was made, with a profound stage ≥ IIIC1. The patient was further treated and staged with abdominal hysterectomy with bilateral salpingo-oophorectomy, para-aortic lymphadenectomy and infracolic omentectomy. Figure 1 is an intraoperative image that demonstrates the final surgical anatomy after the performance of comprehensive para-aortic lymphadenectomy.

Follow-up and outcomes

The final histopathological diagnosis was a mixed high-grade carcinoma of the LUS, 85% serous carcinoma, and 5% undifferentiated carcinoma or carcinosarcoma. The carcinoma invaded the cervix and the lower segment of the uterus and expanded to the endometrium and adnexa. The right ovary was invaded also by the tumor, whereas the omentum was intact. Out of the total 23 para-aortic lymph nodes dissected, one of them was invaded by tumor cells. As a result, a final diagnosis of mixed-type endometrial cancer stage IIIC2 was made and the patient was referred, based on a new MTB decision, for chemotherapy, external beam radiation therapy (EBRT), and brachytherapy. The patient initially performed six cycles of carboplatin-taxol every three weeks as per standard protocol, followed by 25 days of EBRT (overall 45 Gy) and three fractions of brachytherapy (overall 10 Gy). All complementary treatments were performed uneventfully, with only mild fatigue mentioned by the patient. The patient is followed up uneventfully both on surgical and oncological levels. The overall survival (OS) is currently 18 months and the disease-free survival (DFS) is 11 months.

## Discussion

The present case report describes the exceptional and rare case of a patient initially diagnosed with an ASC-US cytology and finally diagnosed with a mixed-type, high-risk, stage IIIC2, endometrial cancer. Endometrial carcinoma of the LUS is a poorly described malignancy due to its scarcity, affecting mostly younger females [3]. Clinically, it may be misdiagnosed as endocervical carcinoma since these two clinical entities may present similar clinical manifestations. From a therapeutic perspective, it is crucial to define its origin as treatment is totally different between early-stage endometrial or cervical cancer. Therefore, preoperative imaging with MRI and immunohistochemistry could assist in distinguishing endocervical and endometrial tumors. Indeed, using MRI, Haider et al. found that endometrial hypertrophy, endometrial tumor, tumor advancement in the uterus, and muscular invasion via the endometrium occurred at a significantly higher frequency in endometrial cancer than in cervical adenocarcinoma and that these characteristics were useful for discriminating between the two conditions [4]. On the contrary, Westin et al. found that MRI was not necessarily useful for preoperative discrimination [5]. Furthermore, endometrial adenocarcinomas express estrogen receptors and vimentin while they are negative for carcinoembryonic antigen (CEA), which is conversely expressed in endocervical carcinomas [6].

The use of p16 immunohistochemistry and HPV in situ hybridization may be decisive for the diagnosis of endocervical carcinoma [7]. It is known that HPV DNA detection and p16 overexpression occur in cervical adenocarcinoma at a higher frequency than in endometrial cancer. Expression of p16 protein is also observed in endometrial cancer based on immunostaining and may actually have a significant prognostic impact. Comparison of immunostaining results indicated diffuse p16 overexpression in cervical cancer, but spotted and weak expression in endometrial cancer, and this may allow discrimination between the two diseases [8].

Conization before radical hysterectomy, which was also performed in the present case, seems to be beneficial in patients who underwent minimally invasive surgery (MIS), with negative pelvic lymph nodes and tumor size less than 4 cm, and is associated with a lower risk of recurrence and better five-year overall survival [9]. Surgery represents the current mainstay of treatment and an important part of staging procedures as far as endometrial cancer is concerned. Primary treatment of high-risk endometrial cancer consists of total hysterectomy, and bilateral salpingo-oophorectomy with pelvic and/or para-aortic lymphadenectomy [10]. According to the latest European Society of Medical Oncology (ESMO) - European Society of Gynecologic Oncology (ESGO) consensus, surgical lymph node staging is crucial in high-risk/intermediate cases in order to estimate the extent of the disease and guide the follow-up treatment of the patient, regarding the adjuvant therapy [10]. In our case, additional para-aortic lymphadenectomy was performed as, according to a recent meta-analysis, combined pelvic and para-aortic lymphadenectomy is associated with critical survival benefits and lower need for radiotherapy, in the context of five-year OS and five-year DFS rate, significantly higher three-year OS rate and essentially improved overall survival and disease-free survival hazard risk [11].

According to the American Society for Colposcopy and Cervical Pathology (ASCCP) guidelines, the management of ASC-US cytology includes either a repeat of the cytology test in 12 months or the performance of an HPV test. Colposcopy may also be performed pending the results of the aforementioned examinations. However, in the case of an ASC-H result, colposcopy is primarily indicated as appropriate management, just as performed in our case.

The literature demonstrates controversial results regarding the high-grade Squamous Intraepithelial Lesion (HGSIL) Cervical intraepithelial neoplasia (CIN) II/CIN III rate of ASC-US cytology. It has been reported a rate ranging from 0.7 to 41.1% but no definitive conclusions regarding the exact rate of ASC-US progression to HGSIL may be made as various studies present conflicting methodology and different follow-up strategies [12,13]. More specifically, in a cohort of 154 patients, Fanny et al. reported an HGSIL rate of 21.4% [12]. Similar results were also indicated by Tropé et al. [13], who reported an overall rate of 23.2% in a retrospective cohort of 625 cases, and by Petousis et al. [14], reporting a rate of 28.4% in a prospective cohort of 134. Nonetheless, there are reports of remarkably lower HGSIL rates. Emerson et al. [15] noted only 6.0% of 643 cases progressed to HGSIL after a 9-year follow-up, while there are also published studies describing even rates of 0.7%. Taking into account previous results, clinicians must be extremely aware of the possible development of HGSIL in patients with ASC-US.

This case report definitely presents certain limitations. First of all, it is a case admitted to us after an initial cytological diagnosis of ASC-US for which the Pap smear was presented elsewhere. Furthermore, the initial work-up before the cervical biopsy was also performed elsewhere under the guidance of a non-certified physician. However, the main management of the case was performed by a dedicated unit, demonstrating a case in which, despite the initial mild cytological diagnosis, a high-risk endometrial cancer was underlying. This case is rather educative to demonstrate that careful diagnostic work-up is needed in such challenging cases in order to identify the proper disease and finally optimize the treatment plan. To our knowledge, this is potentially one among very few described cases of ASC-US cytology finally associated with high-risk endometrial cancer originating from LUS.

## Conclusions

This case report describes one among a few published cases of high-risk, mixed-type, advanced-stage endometrial cancer originating from the lower uterine segment that was initially presented with ASC-US cytology. Cautious diagnostic and therapeutic management permitted the optimal treatment of this rather unusual case. Optimal approach and treatment of the oncological patient from certified experts in dedicated departments may actually maximize the possibility of favorable survival outcomes.
